# *β-*CD Dimer-immobilized Ag Assembly Embedded Silica Nanoparticles for Sensitive Detection of Polycyclic Aromatic Hydrocarbons

**DOI:** 10.1038/srep26082

**Published:** 2016-05-17

**Authors:** Eunil Hahm, Daham Jeong, Myeong Geun Cha, Jae Min Choi, Xuan-Hung Pham, Hyung-Mo Kim, Hwanhee Kim, Yoon-Sik Lee, Dae Hong Jeong, Seunho Jung, Bong-Hyun Jun

**Affiliations:** 1 Department of Bioscience and Biotechnology, Konkuk University, Seoul 143-701, Republic of Korea; 2 Department of Chemistry Education, Seoul National University, Seoul 151-742, Republic of Korea; 3 School of Chemical and Biological Engineering, Seoul National University, Seoul 151-742, Republic of Korea

## Abstract

We designed a *β*-CD dimer on silver nanoparticles embedded with silica nanoparticles (Ag@SiO_2_ NPs) structure to detect polycyclic aromatic hydrocarbons (PAHs). Silica NPs were utilized as a template for embedding silver NPs to create hot spot structures and enhance the surface-enhanced Raman scattering (SERS) signal, and a thioether-bridged dimeric *β*-CD was immobilized on Ag NPs to capture PAHs. The assembled Ag NPs on silica NPs were confirmed by TEM and the presence of *β*-CD dimer on Ag@SiO_2_ was confirmed by UV-vis and attenuated total reflection-Fourier transform infrared spectroscopy. The *β*-CD dimer@Ag@SiO_2_ NPs were used as SERS substrate for detecting perylene, a PAH, directly and in a wide linearity range of 10^−7^ M to 10^−2^ M with a low detection limit of 10^−8^ M. Also, the *β*-CD dimer@Ag@SiO_2_ NPs exhibited 1000-fold greater sensitivity than Ag@SiO_2_ NPs in terms of their perylene detection limit. Furthermore, we demonstrated the possibility of detecting various PAH compounds using the *β*-CD dimer@Ag@SiO_2_ NPs as a multiplex detection tool. Various PAH compounds with the NPs exhibited their distinct SERS bands by the ratio of each PAHs. This approach of utilizing the assembled structure and the ligands to recognize target has potential for use in sensitive analytical sensors.

Polycyclic aromatic hydrocarbons (PAHs) consist of several benzene rings and are carcinogens, teratogens and mutagens^1^. The sources of these compounds are diverse as they are byproducts of incomplete combustion of fossil fuels or organic substances. Various PAH detection methods have been developed. Generally, PAHs in water, soil and solid waste are analyzed after extraction and concentration by chromatography such as, liquid chromatography with fluorescence detection (LC/FLDs)^2^, high-performance liquid chromatography (HPLC) with UV and fluorescence^3^,^4^,^5^, gas chromatography coupled with a flame ionization detector (GC/FlD)^6^ and mass spectrometry (GC/MS)^7^,^8^. The PAH detection limits of these techniques are in the ppb range. However, the chromatography method is hampered by the prolonged sample preparation required^9^. Also, application is problematic because of their incompatibility with column chromatography. Surface-enhanced Raman spectroscopy (SERS) has been proposed to overcome these problems^10^,^11^.

SERS is an ultra-sensitive analytical technique^12^,^13^, which can detect very low concentrations of the target compound^14^,^15^ and determine structural information^16^. Over the past decade, a range of multidirectional strategies for detecting chemicals on SERS substrates have been investigated, utilizing single-metal NPs with a modified surface and various shapes^17^,^18^,^19^,^20^ and sol-gels^21^ as sensors. Further, metal NPs held together in solution can generate hot spots^22^,^23^, which have been used for SERS detection. However, aggregation of metal NPs in solution is difficult to control due to their instability and uncontrollability. Assembled Ag SERS substrates comprising a silica core and Ag NPs could overcome the weaknesses mentioned above, affording high sensitivity^24^. However, Ag NPs on the surface of silica core particles have poor affinity to PAHs.

Cyclodextrins (CDs), which have hydrophobic internal holes, are excellent receptors of PAHs and can be placed on the metal surface via the hydrophobic effect^25^,^26^. In particular, bridged dimeric *β*-CDs were reported to exhibit significantly enhanced binding and recognition of target materials due to cooperative binding of two neighboring CDs, compared to native CDs^27^,^28^,^29^,^30^. However, these bridged dimeric *β*-CDs are not frequently utilized for sensing applications and their use for detection of PAHs has not been investigated extensively to date^31^.

In this study, we used *β*-CD dimers-immobilized Ag assembled silica NPs (*β*-CD dimer@Ag@SiO_2_ NPs) as a SERS substrate to detect PAHs. The assembled Ag structures enhanced the SERS signals and the *β*-CD dimers functioned as affinity ligands for PAHs. The structure the of *β*-CD dimer@Ag@SiO_2_ NPs was analyzed by transmission electron microscopy (TEM), UV-vis spectroscopy and attenuated total reflection-Fourier transform infrared spectroscopy (ATR-FTIR). Sensitivity of *β*-CD dimer@Ag@SiO_2_ NPs for detecting PAHs was compared with that of Ag@SiO_2_ to determine the role of the *β*-CD dimer. Additionally, the possibility of multiplex detection was tested using mixtures of several PAHs, such as phenanthrene, pyrene and benzo(a)pyrene.

## Results and Discussion

### Characterization of *β*-CD dimer@Ag@SiO_2_ NPs structure

**Figure 1:**
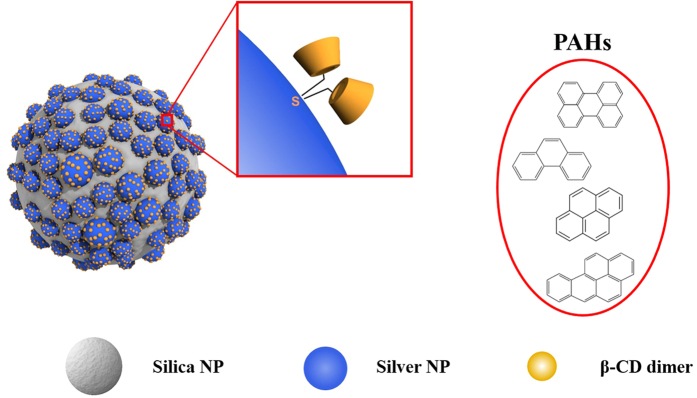
Schematic of *β*-CD dimer-immobilized Ag assembly with embedded silica NPs for detection of PAHs.

The structure of *β*-CD dimer@Ag@SiO_2_ NPs, used as a SERS substrate for detection of PAHs, is shown in Fig. 1. Silica NPs of ~180 nm diameter were prepared according to the Stöber method and utilized as a template to generate assembled Ag NPs on silica NPs. Silica NPs were first thiolated with 3-mercaptopropyl trimethoxysilane (MPTS) to introduce thiol groups to their surface.

Ag NPs were embedded on silica NPs by reduction of silver nitrate by octylamine with polyvinylpyrrolidone (PVP) in ethylene glycol. Thioether-bridged *β*-CD dimer, which was prepared as reported previously^32^, was immobilized on Ag NPs by means of the affinity of thiol groups for silver. Here, *β*-CD dimers were used to capture PAHs so that PAHs can be located on the surface of the Ag NP through the host-guest complexation. The assembled Ag NPs on silica NPs were designed to create hot spots to enhance the SERS signals of target molecules.

**Figure 2:**
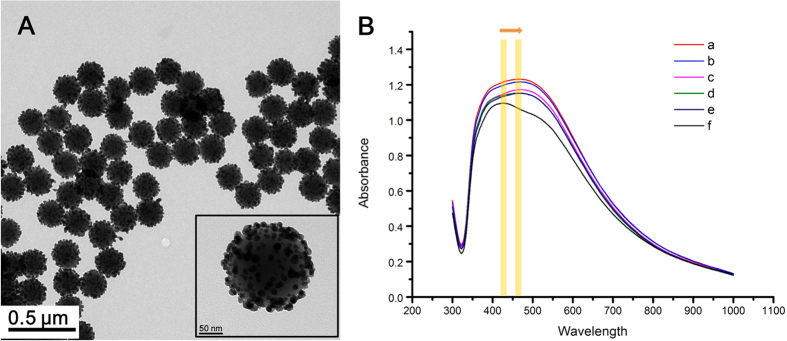
(**A**) TEM images of single Ag@SiO_2_ NPs and distribution of Ag@SiO_2_ NPs in EtOH. (**B**) UV-vis spectra of *β*-CD dimer@Ag@SiO_2_ NPs added perylene at (a) 1 × 10^−4^ M (b) 1 × 10^−5^ M (c) 1 × 10^−6^ M and (d) 1 × 10^−7^ M, and (e) *β*-CD dimer@Ag@SiO_2_ NPs in the absence of perylene and (f) Ag@SiO_2_ NPs.

Figure 2A shows TEM images of the Ag@SiO_2_ NPs. These NPs were uniform in size (*ca.* 206 ± 10 nm) and well dispersed without aggregation. The size of Ag NPs embedded on the surface of silica NPs was 8.4 ± 3.7 nm. *β*-CD dimers were immobilized on the surface of the assembled Ag NPs on the silica NPs to capture PAHs via host-guest interactions. To investigate the optical properties of these particles, UV–vis spectra were recorded (Fig. 2B). The UV-vis spectrum of the assembled Ag@SiO_2_ NPs (line f) showed a broad band from 322 to 800 nm with a maximum peak at 430 nm, indicating the presence of aggregates of Ag NPs on the surface of silica NPs. Following assembly of *β*-CD dimer on the surface of the Ag@SiO_2_ NPs, UV-vis spectrum of the *β*-CD dimer@Ag@SiO_2_ NPs show a slightly shifted localized plasmon resonance band from 430 nm to 464 nm (line e). The spectra of *β*-CD dimer@Ag@SiO_2_ NPs with various concentration of perylene from 1 × 10^−7^ to 1 × 10^−4^ M in line a–d were showed a peak of 452 nm, which is assigned as perylene^33^. The increasing of absorbance peaks by perylene were relatively weak, compared to the absorbance peak of *β*-CD dimer@Ag@SiO_2_, however the intensity of absorbance peaks were increased by raising the concentration of perylene. We believe that when perylene was caught by *β*-CD dimer@Ag@SiO_2_, the presence of perylene on the surface of *β*-CD dimer@Ag@SiO_2_ may have caused the combination effect of the extinction coefficiency of our materials to visible light that increased the intensity of the absorbance peak. To confirm assemble of *β*-CD dimers on the surface of the Ag@SiO_2_ NPs, attenuated total reflection-Fourier transform infrared spectroscopy (ATR-FTIR) was performed. The IR spectra of Ag@SiO_2_ NPs and *β*-CD dimer@Ag@SiO_2_ NPs are shown in Figure S1. The IR spectra of the Ag@SiO_2_ NPs and the *β*-CD dimer@Ag@SiO_2_ NPs differed. The intensity of the band at 2923 cm^−1^, assigned to C-H stretching vibration, increased due to the *β*-CD dimers. Also, a new band at 2349 cm^−1^ was assigned to the *β*-CD dimers. Band shifts from 1048 cm^−1^ to 1044 cm^−1^ and from 1656 cm^−1^ to 1685 cm^−1^ were observed due to the assembly of *β*-CD dimer on Ag@SiO_2_ NPs. In particular, a characteristic band at 708 cm^−1^ was assigned to C-S stretching vibration bands of the *β*-CD dimers. Our result is consistent with a previous report^34^. Therefore, we demonstrated that *β*-CD dimers were successfully assembled on the Ag@SiO_2_ NPs.

### Detection of perylene using *β*-CD dimer@Ag@SiO_2_ NPs

*β*-CD can interact with PAHs in its hydrophobic cavity and form various host–guest complexes^26^,^35^,^36^,^37^,^38^. Unlike native CDs, dimeric *β*-CDs exhibit significantly enhanced binding and recognition of target materials due to cooperative binding of two neighboring CDs^27^,^28^,^29^,^30^. Therefore, we assumed that the *β*-CD dimer-modified Ag@SiO_2_ NPs would facilitate enrichment of PAHs on the metal surface, and PAH–CD complexes could be detected by surface-enhanced Raman spectroscopy.

**Figure 3:**
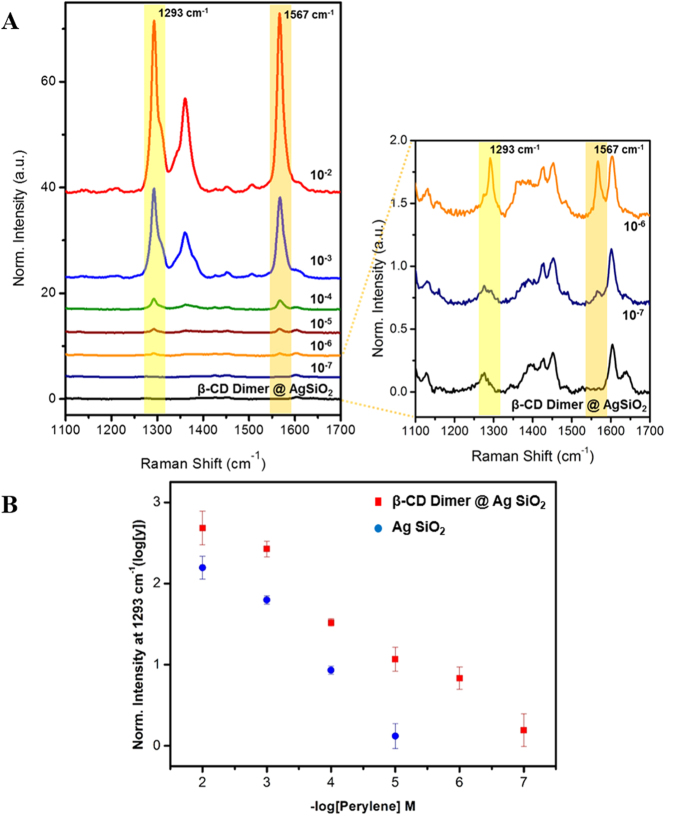
(**A**) SERS spectra of *β*-CD dimer@Ag@SiO_2_ NPs at 1 × 10^−2^ M to 1 × 10^−7^ M. (**B**) Normalized SERS intensities of *β*-CD dimer@Ag@SiO_2_ and Ag@SiO_2_ with perylene at 1293 cm^−1^.

SERS spectra of *β*-CD dimer@Ag@SiO_2_ NPs treated with 1 × 10^−2^ M to 1 × 10^−7^ M perylene for 30 min are shown in Fig. 3. The *β*-CD dimer@Ag@SiO_2_ NPs were mixed with perylene and the SERS signals were measured in a capillary tube. SERS intensity increased with perylene concentration, particularly the peaks at 1293 cm^−1^ and 1576 cm^−1^. The peak at 1293 cm^−1^ was assigned to the C-C stretching on bridges and the peak at 1576 cm^−1^ was assigned to the C-C stretching on benzene rings of perylene^39^,^40^. Perylene at up to 10^−7^ M was detected by *β*-CD dimer@Ag@SiO_2_ NPs using the capillary method and 10^−8^ M using the drop-cast method (Figure S2). To confirm the *β*-CD dimer effect, the Ag@SiO_2_ NPs lacking *β*-CD dimers were mixed with perylene. The SERS spectra of the Ag@SiO_2_ NPs in the presence of perylene are shown in Figure S3. The normalized SERS intensity at 1293 cm^−1^ of the *β*-CD dimer@Ag@SiO_2_ NPs and Ag@SiO_2_ NPs with perylene in various concentrations are shown in Fig. 3B. The SERS signals of the *β*-CD dimer@Ag@SiO_2_ with the same concentration of perylene were markedly higher than those of Ag@SiO_2_ NPs lacking *β*-CD dimers. A similar phenomenon appeared at 1567 cm^−1^ (Figure S4). Therefore, we concluded that *β*-CD dimers played an important role in capturing and enriching PAHs on the surface of the Ag@SiO_2_ NPs. Although the detection limit of perylene using the SERS signals was equivalent to those of traditional methods, such as isotope dilution capillary column gas chromatography/mass spectrometry (GC/MS) and high-performance liquid chromatography (HPLC), the SERS technique is more cost-effective and less time-consuming.

### Multiplex detection of PAHs using *β*-CD dimer@Ag@SiO_2_ NPs

To utilize the multiplex detection capability of the *β*-CD dimer@Ag@SiO_2_ NPs for PAH compounds, phenanthrene, benzo(a)pyrene, pyrene and perylene were mixed with *β*-CD dimer@Ag@SiO_2_ NPs.

**Figure 4:**
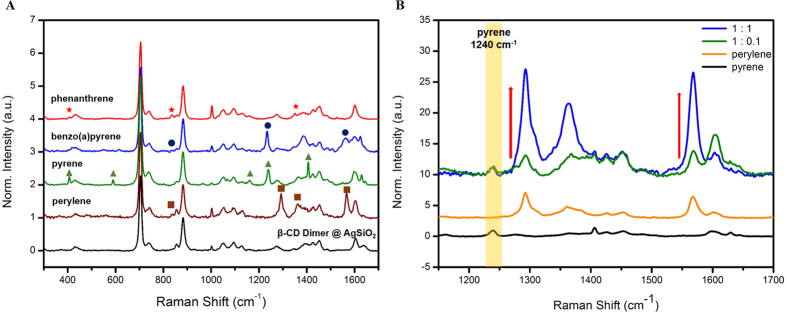
(**A**) SERS spectra of *β*-CD dimer@Ag@SiO_2_ NPs with four PAHs. (**B**) SERS spectra of *β*-CD dimer@Ag@SiO_2_ NPs in the presence of the indicated concentrations of perylene and a fixed concentration of pyrene.

Figure 4A shows the SERS spectra of phenanthrene, benzo(a)pyrene, pyrene and perylene (10^−4^ M each) mixed with *β*-CD dimer@Ag@SiO_2_ NPs. The SERS bands of the *β*-CD dimer@Ag@SiO_2_ NPs in the presence of various PAHs differed from those of *β*-CD dimer@Ag@SiO_2_ NPs only. In particular, pyrene showed a characteristic peak at 1240 cm^−1^ and perylene a characteristic peak at 1293 cm^−1^. Benzo(a)pyrene had a characteristic peak at 1234 cm^−1^. Phenanthrene exhibited somewhat weaker intensity than the other PAHs, and its characteristic peak was at 836 cm^−1^. These results demonstrate that *β*-CD dimer@Ag@SiO_2_ NPs can interact with various PAHs compounds and show distinct SERS bands. More experiments to provide the sensor selectivity were performed. Each organic compound (perylene, naphthalene, toluene, isopropyl acid and ethylene glycol) at the concentration of 10^−4^ M was added in *β*-CD dimer@Ag@SiO_2_ NPs solutions and those solutions were compared with *β*-CD dimer@Ag@SiO_2_ NPs solution. Perylene showed its characteristic peak, while other solutions that had added organic compounds did not show their peaks in Raman spectra.

Figure S5 (A) showed the Raman spectra of those solutions. The presence of interferences showed insignificant changes of spectra compared to that of no targeted sample. To investigate the interference of organic compounds in detail, toluene was chosen as a model for investigating the effect of interference at high concentration. The perylene/toluene ratios were adjusted to 1:1 (10^−4^ M : 10^−4^ M) and 1:10 (10^−4^ M : 10^−3^ M). *β*-CD dimer@Ag@SiO_2_ NPs was added in a mixed solution of perylene and toluene. At a perylene/toluene ratio of 1:1, the mixture showed the characteristic peak of perylene at 1567 cm^−1^. However, at a perylene/toluene ratio of 1:10, the mixed solution appeared in the peaks of toluene (10^−3^ M) at 622, 785 and 1030 cm^−1^ and perylene (10^−4^ M) at 1567 cm^−1^ in Raman spectra before washing. After washing, these peaks of toluene disappeared. The intensity at 1567 cm^−1^ in Figure S5 (B) showed insignificant change in the presence of toluene. This result demonstrated that *β*-CD dimer@Ag@SiO_2_ NPs can be a selective sensor.

To investigate the multiplex detection capability, we used pyrene and perylene as model PAHs for SERS detection. *β*-CD dimer@Ag@SiO_2_ NPs were mixed with a mixture of pyrene (10^−3^ M) and perylene (1 × 10^−3^ to 1 × 10^−4^ M). Figure 4B shows an average Raman spectrum of the *β*-CD dimer@Ag@SiO_2_ NPs in the presence of pyrene and perylene. As the perylene concentration increased, the intensities of the characteristic peaks of perylene at 1293 cm^−1^ and at 1567 cm^−1^ also increased, while the typical peak of pyrene at 1240 cm^−1^ did not change markedly. Therefore, *β*-CD dimer@Ag@SiO_2_ NPs could be used for multiplex detection of pyrene and perylene.

To demonstrate the stability of our sensor, we measured the intensity of perylene at 1567 cm^−1^ after several washing steps. Acetonitrile, which was mentioned in the previous report for extracting PAHs in HPLC technique^41^,^42^, was used as a solvent for washing.

Figure S6 showed the Raman spectra of *β*-CD dimer@Ag@SiO_2_ in the 10^−4^ M perylene solution after each washing (Figure S6 (A)). The characteristic peak at 1567 cm^−1^ was plotted in Figure S6 (B). After it was washed 5 times, the intensity of the perylene peak at 1567 cm^−1^ was insignificantly changed. Perylene with the *β*-CD dimer@Ag@SiO_2_ was stable after washing 5 times.

We prepared *β*-CD dimer-immobilized Ag assembly embedded silica NPs (*β*-CD dimer@Ag@SiO_2_ NPs). The Ag@SiO_2_ NPs were uniform in diameter (*ca.* 206 ± 10 nm) with 8.4 ± 3.7 nm Ag NPs on their surface. The *β*-CD dimer@Ag@SiO_2_ NPs were able to detect perylene selectively at concentrations of 1 × 10^−2^ to 10^−7^ M. The limit of detection was 10^−8^ M. The *β*-CD dimer@Ag@SiO_2_ NPs can be used for multiplex detection of PAHs compounds, such as pyrene, perylene and so on. This method of combining an assembled structure and the ligands to recognize target materials has considerable potential for detection of chemicals or biomolecules.

## Method

### Preparation of assembly Ag-embedded silica nanoparticles (Ag@SiO_2_ NPs)

Approximately 180 nm Ag assembly-embedded silica NPs were synthesized using a method reported previously^24^,^43^ First, silica NPs were prepared using the Stöber method and their surface was modified with thiol groups. Forty milliliters of 99.9% EtOH, 3 mL NH_4_OH, and 1.8 mL TEOS were mixed and the solution stirred vigorously for 20 h at 25 °C. Then, the mixture was centrifuged, washed with 95% EtOH, dispersed in absolute EtOH, and the volume adjusted to 50 mg mL^−1^ silica NPs solution. Two hundred microliters of MPTS and 50 μL NH_4_OH were added to 4 mL silica NPs solution (50 mg mL^−1)^ and the mixture was stirred vigorously for 12 h at 25 °C. The solution was centrifuged, followed by washing with EtOH to remove excess reagent. Second, Ag NPs were embedded on the surface of thiolated silica NPs by reducing silver nitrate with octylamine and PVP in ethylene glycol. Five milligrams of PVP in 25 mL EG and 26 mg AgNO_3_ in 25 mL EG were added to a thiolated silica NP (30 mg) suspension (final AgNO_3_ concentration, is 15 mM). After thorough mixing, 41.4 μL octylamine were added and the resulting suspension was stirred for 1 h at 25 °C. The resulting suspension was centrifuged, washed several times with EtOH, and dispersed in 3 mL absolute EtOH to obtain a 10 mg mL^−1^ Ag@SiO_2_ solution.

### Synthesis of *β*-cyclodextrin dimer

*β*-Cyclodextrin dimer was synthesized according to a reported method^32^. Mono-6-O-p-toluenesulfonyl-*β*-CD (1.00 g, 0.78 mmol), synthesized from *β*-CD by modifying a 6-hydroxy group on the primary side, was dissolved in anhydrous DMF (40 mL) with sodium iodide (1.20 g, 8 mmol) under nitrogen stirring for 5 h at 90 °C. Acetone (25 mL) was added to obtain a precipitate, and the solvent was removed through reduced pressure (rotary evaporator) after filtration and washing. The resulting solid was dissolved in demineralized water (50 mL) and excess acetone was added to induce precipitation. After filtering and drying, the precipitate, which is 6-iodo-6-deoxy-*β*-CD (0.88 g, 0.71 mmol), was dissolved in 8 mL of dry DMF. The resulting solution was reacted with sodium sulfide (27.64 mg, 0.35 mmol) for 15 h at 80 °C. The reaction mixture was precipitated with 200 mL of acetone after concentration under reduced pressure. The precipitates collected on a glass filter were dried under reduced pressure. Final *β*-CD was purified using a Bio-Gel P6 column after dissolving the precipitates in water. The dried final product was obtained with a yield of 13.5% from *β*-CD.

### Preparation of *β*-CD dimer@Ag@SiO_2_ NPs

To *β*-cyclodextrin dimer solution (5 mM in distilled water) was added 5 mg of assembled Ag embedded silica NPs. The suspension was stirred vigorously for 12 h at 25 °C. The suspensions were then centrifuged and washed with EtOH, and redispersed in 1 mL absolute EtOH.

### Interactions of *β*-CD dimer@Ag@SiO_2_ NPs with PAHs

A perylene stock solution (100 mM) was prepared using dichloromethane as the solvent. Subsequently, a series of PAH solutions of various concentrations were obtained by dilution with dichloromethane. The prepared *β*-CD dimer@Ag@SiO_2_ NPs (0.5 mg in 500 μL of EtOH) were mixed with 500 μL of dichloromethane solution containing various concentrations of PAHs and shaken for 30 min under ambient conditions.

### Raman measurements

To evaluate the sensitivity of the synthesized SERS materials, the SERS signals were measured in a capillary tube using a DXR™ Raman Microscope system (Thermo Fisher Scientific, USA). The SERS signals were collected in a back-scattering geometry using a ×10 objective lens. A 532 nm diode-pumped solid-state laser was used as the photo-excitation source, with 10 mW laser power at the sample. Selected sites were measured randomly, and all SERS spectra were integrated for 5 s. The spot size of the laser beam was ~2 μm.

## Additional Information

**How to cite this article**: Hahm, E. *et al*. *β*-CD Dimer-immobilized Ag Assembly Embedded Silica Nanoparticles for Sensitive Detection of Polycyclic Aromatic Hydrocarbons. *Sci. Rep.* **6**, 26082; doi: 10.1038/srep26082 (2016).

## Supplementary Material

Supplementary Information
