# Supplementary material for: β-CD Dimer-immobilized Ag Assembly Embedded Silica Nanoparticles for Sensitive Detection of Polycyclic Aromatic Hydrocarbons
Source: Sci Rep. 2016 May 17;6:26082. doi: 10.1038/srep26082 (PMC4869113; doi:10.1038/srep26082)
Supplement: Supplementary Information [file srep26082-s1.doc]

***β*-CD Dimer-immobilized Ag Assembly Embedded Silica Nanoparticles for** **Sensitive Detection of Polycyclic Aromatic Hydrocarbons**

Eunil Hahm,^a^ Daham Jeong,^a^ Myeong Geun Cha,^b^ Jae Min Choi,^a^ Xuan-Hung Pham,^a^ Hyung-Mo Kim,^a^ Hwanhee Kim,^a^ Yoon-Sik Lee,^c^ Dae Hong Jeong,^b^ Seunho Jung,^a*^ Bong-Hyun Jun ^a*^

^a^ Department of Bioscience and Biotechnology, Konkuk University, Seoul 143-701, Republic of Korea,

^b^ Department of Chemistry Education, Seoul National University, Seoul 151-742, Republic of Korea

^c^ School of Chemical and Biological Engineering, Seoul National University, Seoul 151-742, Republic of Korea

**Name of Corresponding Authors:** Bong-Hyun Jun, Ph.D, Seunho Jung, Ph.D

Tel.: +82-2-450-0521, Fax: +82-2-3437-1977,

E-mail: bjun@konkuk.ac.kr (B.-H. Jun), [shjung@konkuk.ac.kr](mailto:shjung@konkuk.ac.kr), (S. Jung)

**Figure S1**. ATR-FTIR spectra of *β*-CD dimer@Ag@SiO_2_ NPs, Ag@SiO_2_ NPs and *β*-CD dimer. These materials were measured in the solid state.


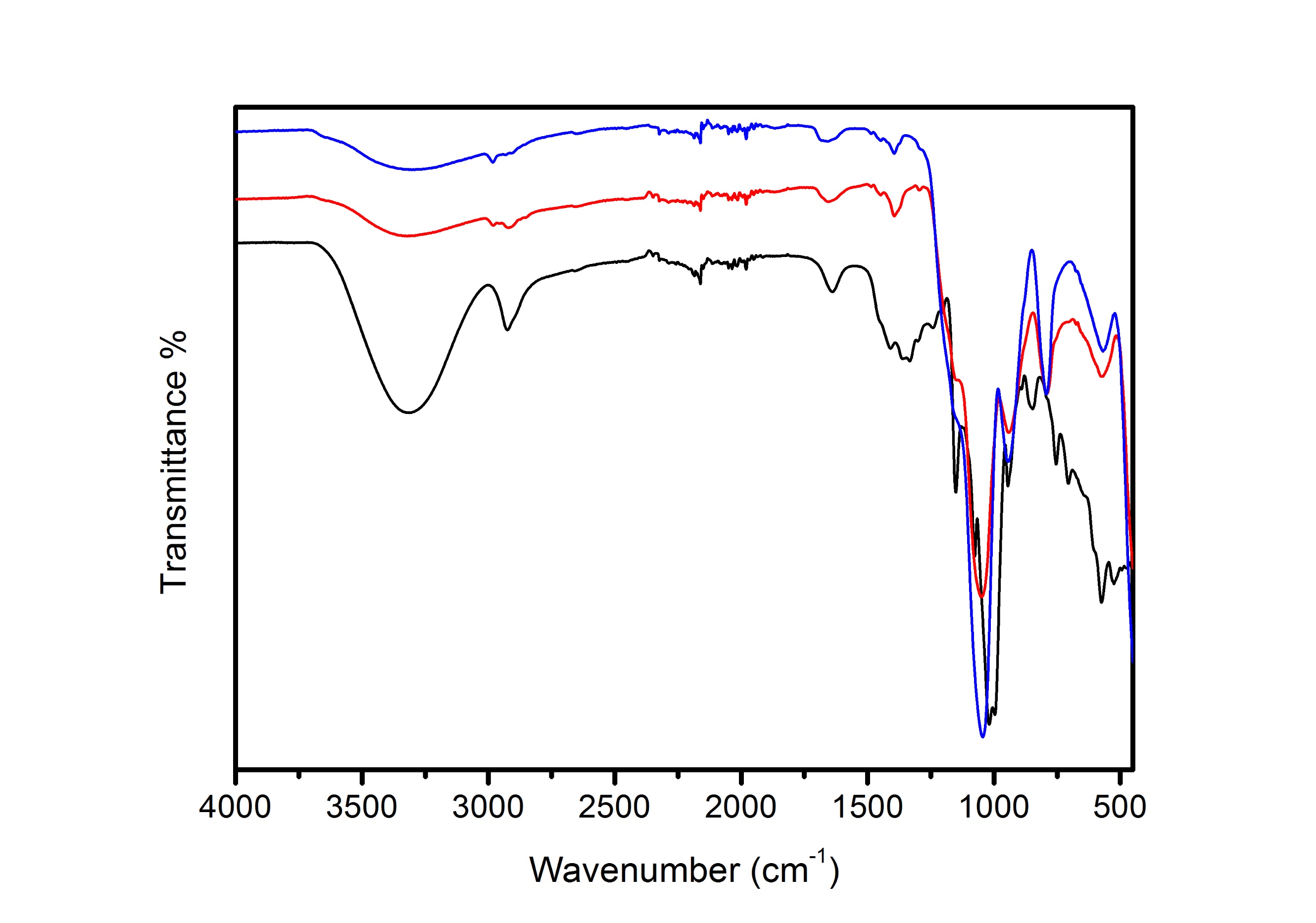

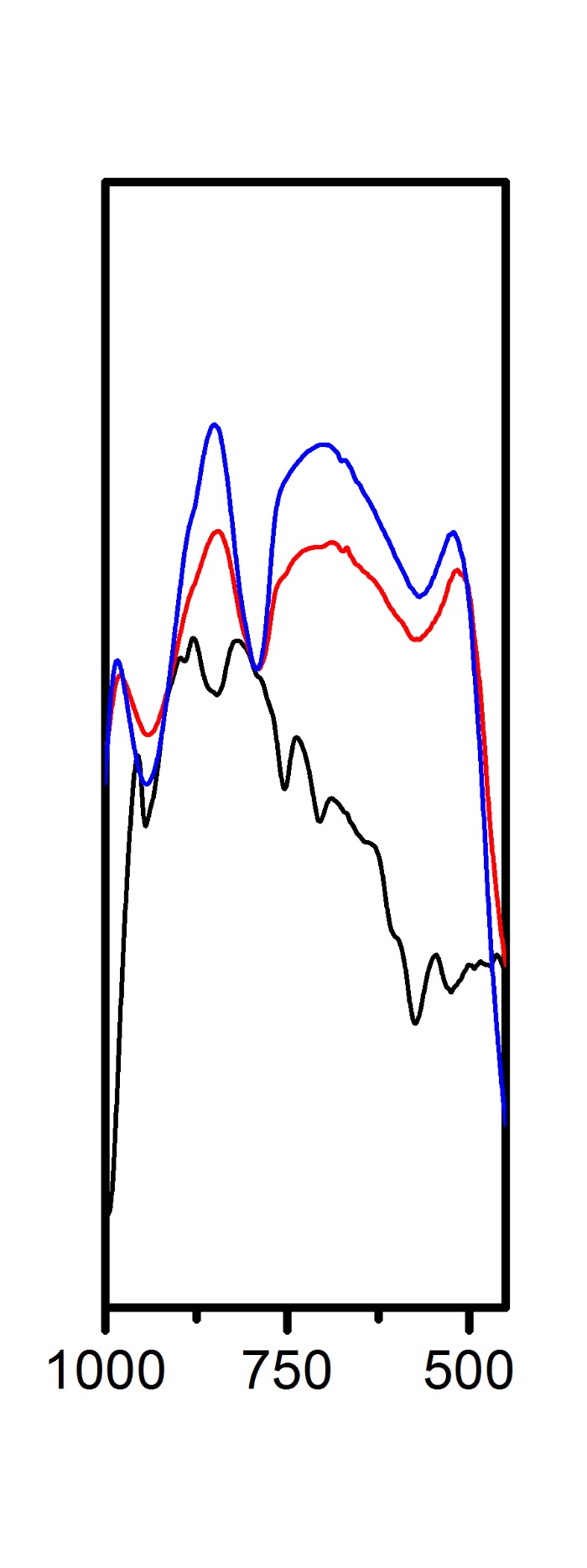

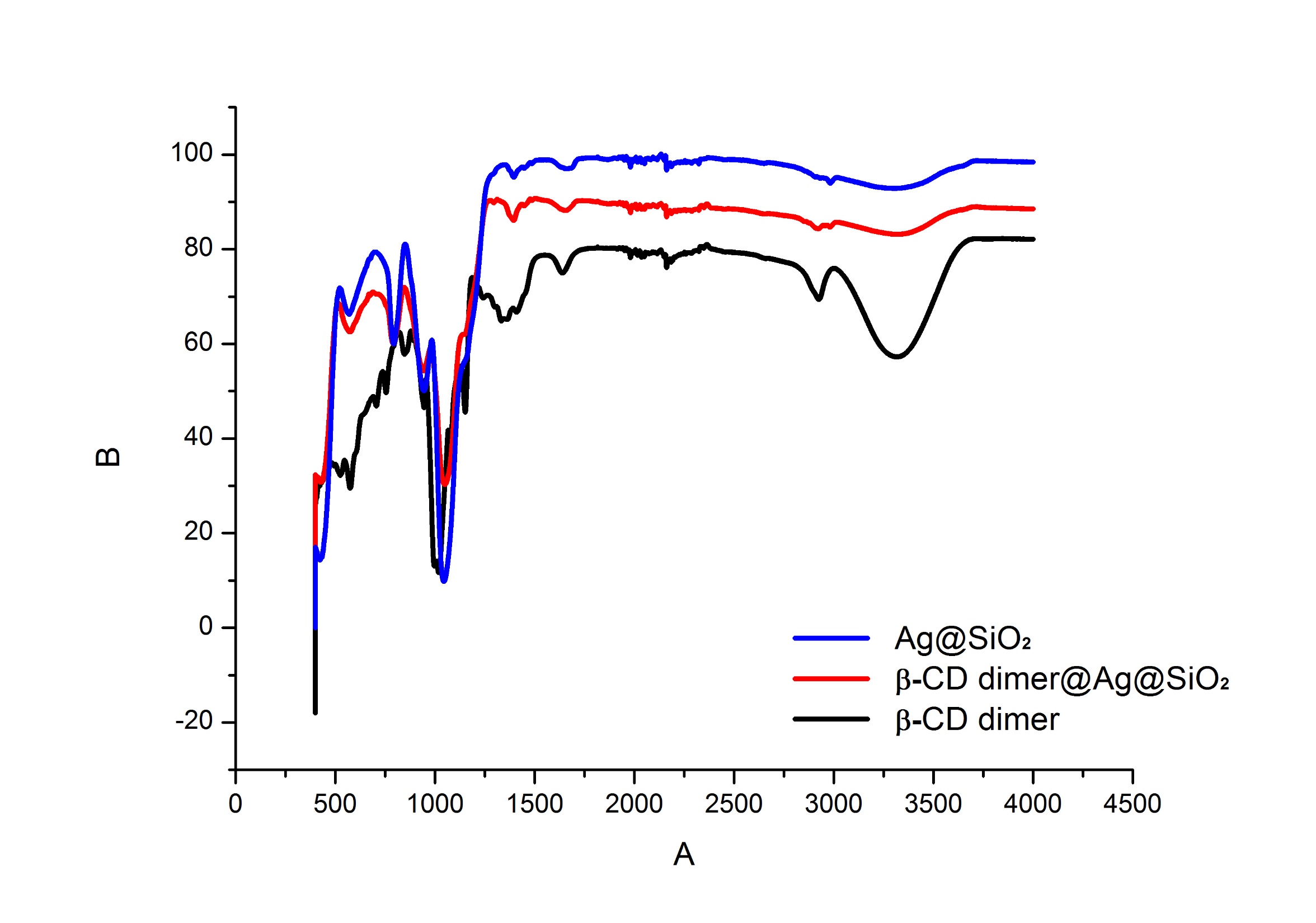


**
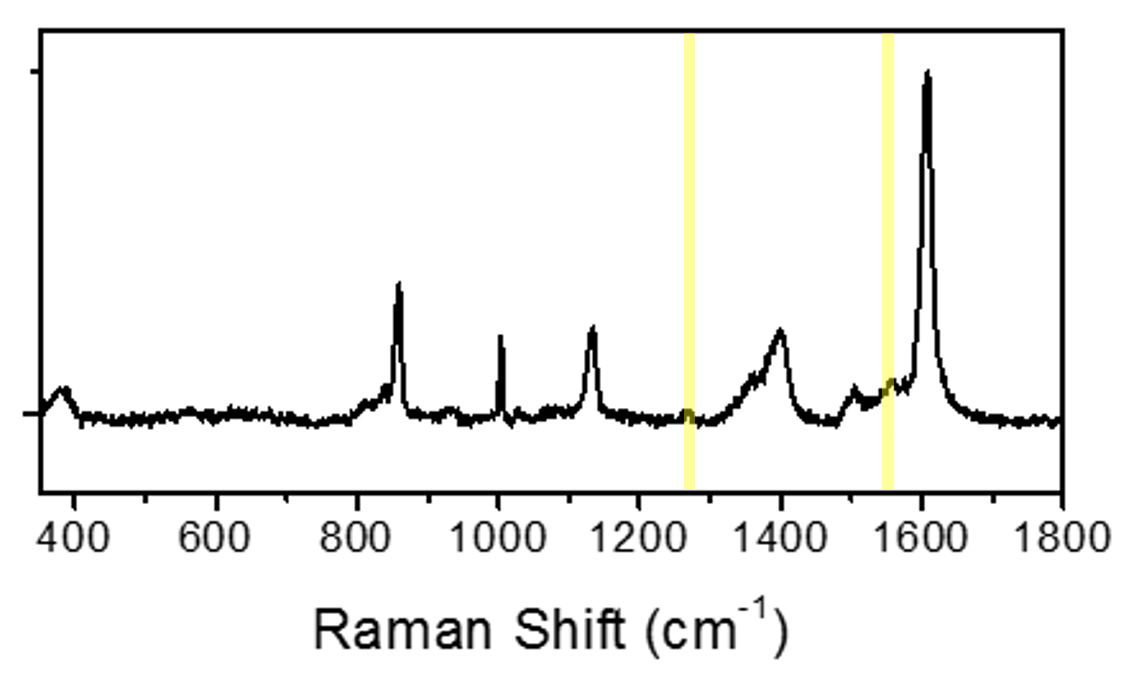
**

**Figure S2.** SERS spectra of perylene at 10^-8^ M using the drying solid method.


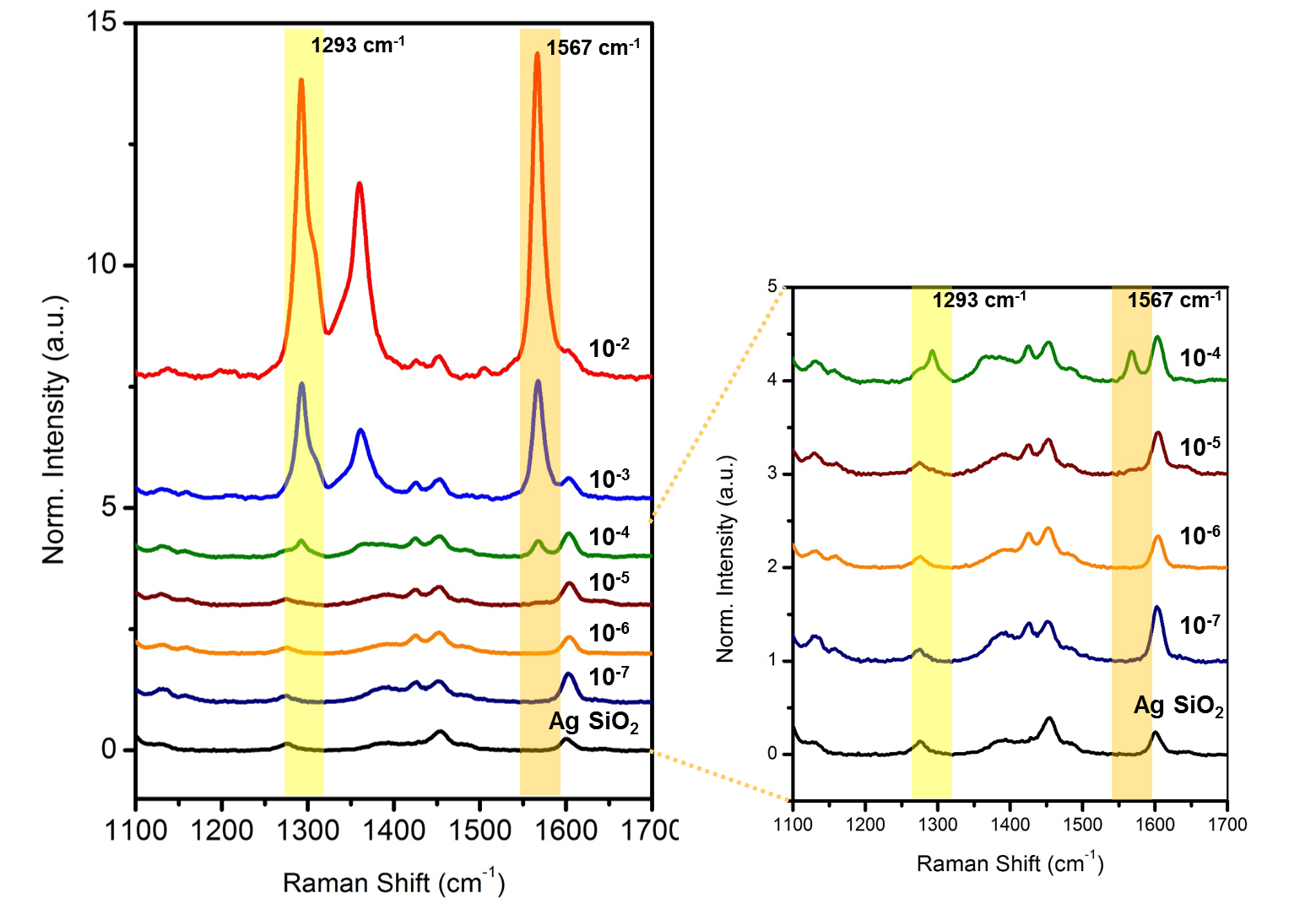


**Figure S3.** SERS spectra of Ag@SiO_2_ NPs at 1 × 10^-2^ M to 1 × 10^-7^ M.


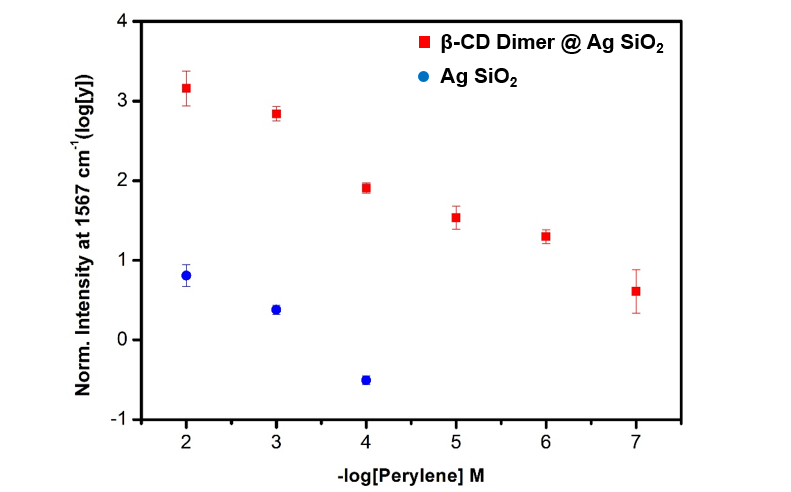


**Figure S4.** Normalized SERS intensity of *β*-CD dimer@Ag@SiO_2_ and Ag@SiO_2_ with perylene at 1567 cm^-1^


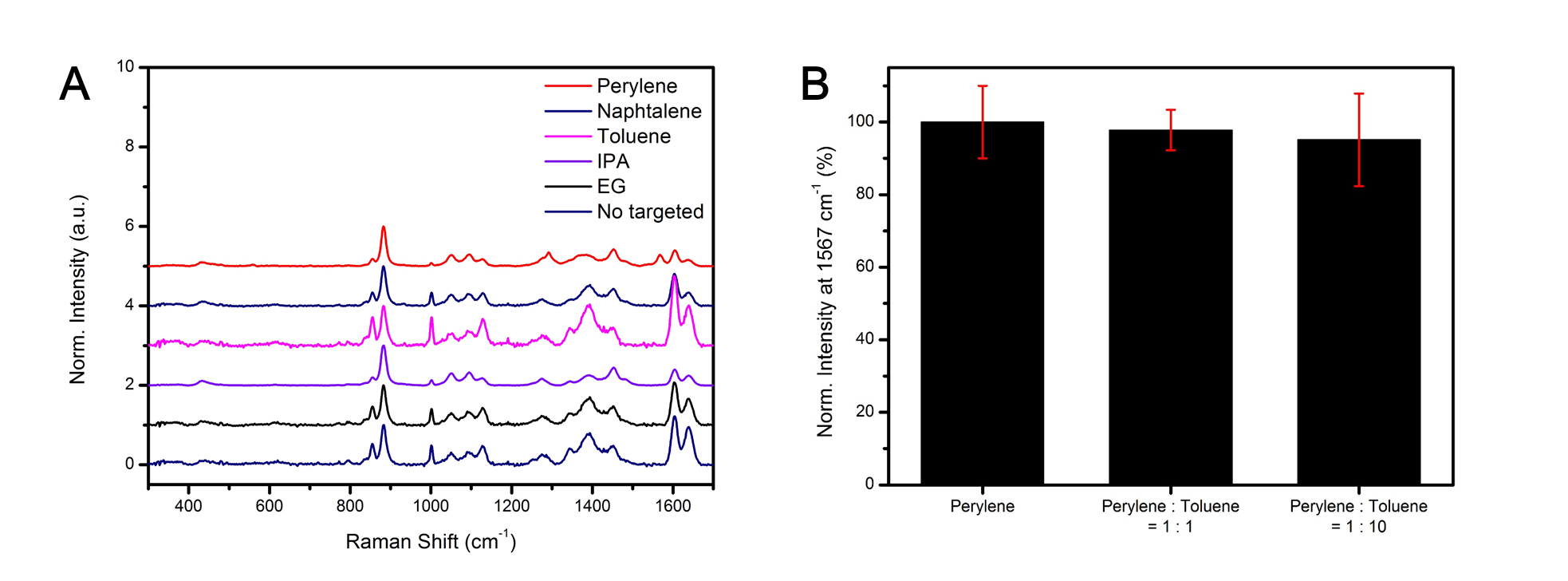


**Figure S5.** (A) The Raman Spectra of ß-CD dimer@Ag@SiO_2_ NPs added each organic compound (B) The Raman intensity of ß-CD dimer@Ag@SiO_2_ NPs added a mixture of perylene and toluene. The perylene/toluene ratio was adjusted to 1:1 and 1:10 at at 1567 cm^-1^.


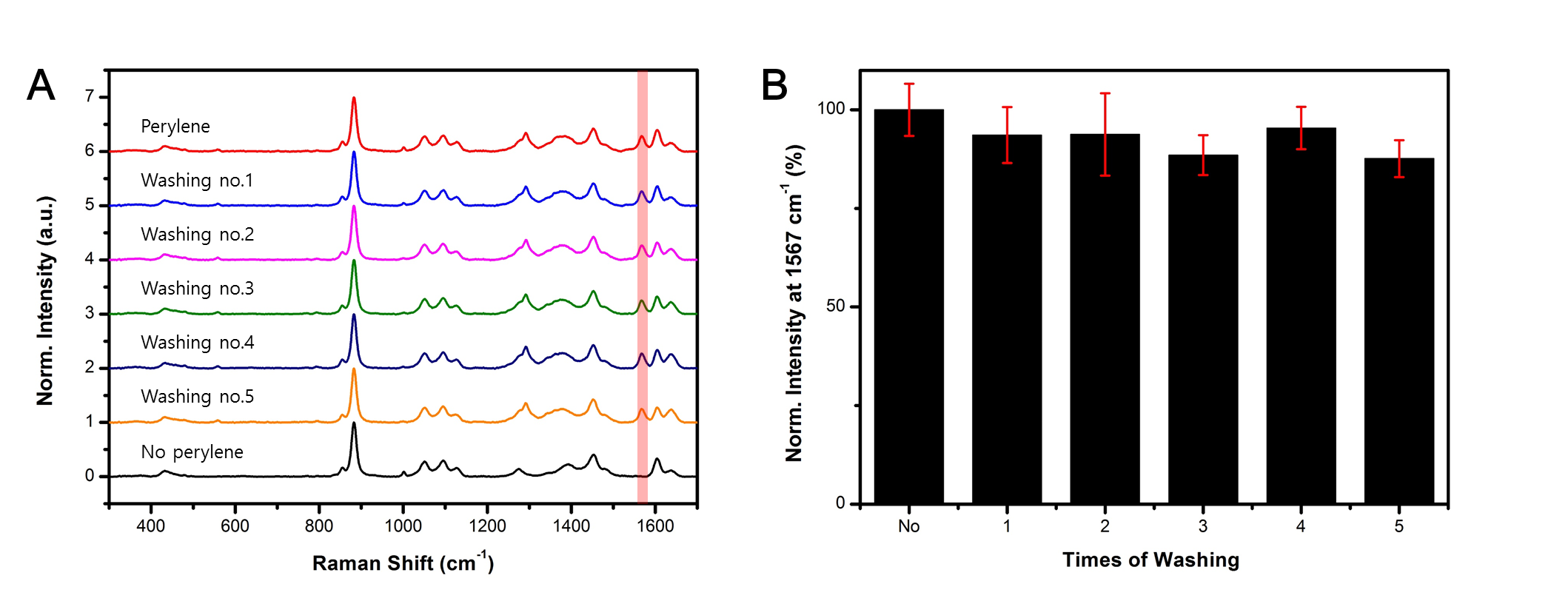


**Figure S6.** Raman (A) spectra and (B) intensity of ß-CD dimer@Ag@SiO_2_ added 10^-4^ M perylene at 1567 cm^-1^ after washing with acetonitrile.
